# Identification, methylation profiling, and expression analysis of stress-responsive cytochrome P450 genes in rice under abiotic and phytohormones stresses

**DOI:** 10.1080/21645698.2021.1908813

**Published:** 2021-04-20

**Authors:** Muhammad Waseem, Feiyan Huang, Qiyu Wang, Mehtab Muhammad Aslam, Farhat Abbas, Fiaz Ahmad, Umair Ashraf, Waseem Hassan, Sajid Fiaz, Xianwen Ye, Lei Yu, Yanguo Ke

**Affiliations:** a College of Horticulture, South China Agricultural University, P.R. China; b College of Agriculture and Life Sciences, Yunnan Urban Agricultural Engineering & Technological Research Centre, Kunming University, Kunming China; c College of Life Sciences, Joint International Research Laboratory of Water and 5 Nutrient in Cops, Fujian Agriculture and Forestry University, Fuzhou, Fujian China; d College of Forestry and Landscape Architecture, South China Agricultural University, Guangzhou P.R. China; e State Key Laboratory of Crop Genetics and Germplasm Enhancement, Nanjing Agricultural University , Nanjing PR China; f Department of Botany, Division of Science and Technology, University of Education Lahore, Punjab, Pakistan; g Institute of Environment and Sustainable Development in Agricultural, Chinese Academy of Agricultural Sciences, Beijing, China; h Department of Plant Breeding and Genetics, University of Haripur, Khyber Pakhtunkhwa, Pakistan; i Kunming Tobacco Corporation of Yunnan Province, Kunming China; j College of Economics and Management, Kunming University, Kunming China

**Keywords:** Abiotic stress, demethylation, DNA-methylation, phytochrome, phylogeny

## Abstract

The cytochrome P450 (CYP) is a large and complex eukaryotic gene superfamily with enzymatic activities involved in several physiological and regulatory processes. As an objective, an *in-silico* genome-wide DNA methylation (5mC) analysis was performed in rice (*Oryza sativa* cv. Zhonghua11), and the epigenetic role of CYPs in two abiotic stresses was observed. Being a stable representative mark, DNA-methylation alters the gene expression under stressful environmental conditions. Rice plants under salinity and drought stresses were analyzed through MeDIP-chip hybridization, and 14 unique genes of the CYP family were identified in the rice genome with varying degrees of methylation. The gene structure, promoter sequences, and phylogenetic analysis were performed. Furthermore, the responses of CYPs to various abiotic stresses, including salinity, drought, and cold were revealed. Similarly, the expression profile of potential CYPs was also investigated under various phytohormone stresses, which revealed the potential involvement of CYPs to hormone regulations. Overall, the current study provides evidence for CYP’s stress regulation and fundamental for further characterization and understanding their epigenetic roles in gene expression regulation and environmental stress regulation in higher plants.

## Introduction

Global climate changes exert a plethora of abiotic stresses on plants, including salinity and drought that ultimately lower crop production.^1,2^ Plants have evolved a variety of physiological and biochemical mechanisms to cope with environmental hazards. However, genomics and molecular biology approaches revealed numerous stress-responsive genes to tolerate the related hazards.^3^ Meanwhile, altering the stress-responsive genes expression level with their protein level helps to understand the plant’s ability to tackle adverse environmental conditions. The Cytochrome P450 (CYP) is an ancient and ubiquitous enzyme protein that is mainly accountable for the oxidative metabolism of diverse exogenous and endogenous compounds.^4^ The CYPs enzymes are designated due to their ability to absorb the light at 450 nm and catalyzed numerous xenobiotic and biosynthetic pathways such as xenobiotics detoxification, carbon assimilation, secondary metabolites biosynthesis, drug deactivation, defense-related compound, UV protection, phytohormones, pigmentation, and procarcinogen activation.^5–9^

Nomenclature and classification of CYPs are based on peptide similarity. According to this system, two CYP proteins sharing a sequence identity of greater than 40% belong to the A-type family, and 55% belong to the non-A-type subfamily. In plants, the A-type was included in *CYP71* clads, whereas the non-A type was grouped in ten clads, including *CYP51/72/74/85/86/97/710/711/727/746*.^10^ Gene duplications and divergence are responsible for this great diversity of CYP gene families under adaptive diversification.^11,12^ In-plant genome, CYPs enzymes are more abundant compared to other organisms. Genome-scale identification of CYP has been performed in numerous model plants, such as 272 members in *Arabidopsis thaliana*, 312 members in *Populus trichocarpa*, 316 members in *Vitis vinifera*, 356 members in *Oryza sativa*, 372 members in *Sorghum bicolor*, and *Glycine max* 322 CYPs.^13–16^ However, the functions of most CYPs are still uncertain. For instance, 30% of CYPs genes are well characterized in Arabidopsis.^17^ CYPs play a pivotal role in lipid biosynthesis, fungal mycotoxins, and flavonoid biosynthesis.^18,19^ A legume-specific CYP 93 C (CYP93C) is a key enzyme of isoflavonoid biosynthesis pathway.^20^ Several cytochromes such as CYP93B/E/G play critical roles as plant secondary metabolites like triterpenoid saponin and flavone biosynthesis.^21,22^ Similarly, CYPs have already been reported in plant organs such as germinating seed, stem, apical bud, and tulip bulbs.^23–25^

5-methylcytosine DNA methylation (5mC) is the most common epigenetic modifier and regulator of their targeted genes. In plants, 5mC and histone modification played vital roles in regulating a variety of physiological processes such as gene expression, genome stability, regulating parental imprinting, and in-plant response to abiotic or biotic stresses.^1,26–29^ However, the global DNA hypomethylation or hypermethylation was also estimated, but their exact mechanism remains unclear.^30–32^ Rice (*O. sativa* L.) is a staple food crop that feeds half the world’s population and is an ideal model plant owing to high sensitivity to salinity and drought, particularly at critical growth stages such as pollen development and seedling stage.^33,34^ Rice plants can cope with environmental hazards by modulating various physiological and biochemical processes. These changes include the changes in enzymatic activities and expression level of different stress-responsive genes involved in salinity, drought, hypoxia, and temperature tolerance.^35^ However, the functional characterization of these genes on behalf of DNA methylation yet needs to be explored.

Previously, we reported the dynamic role of DNA methylation^36^ in regulating stress-responsive zinc-finger protein (ZFP) in rice and found that these genes have a varying degree of methylation.^37^ Wei et al.^38^ reported that the genome-scale identification of CYPs, but their role in rice DNA methylation regulation is still scarce. To avail this opportunity, we identified a total of 14 DNA methylation regulated CYPs in rice. It was observed that these genes are full or partially methylated in different regions and have the potential to tolerate abiotic stresses such as salt, drought, cold, and phytohormones in rice.

## Materials and Methods

### In-silico Identification of CYPs in Rice

5-methylcytosine DNA methylation (5mC) level of CYPs in rice genome was qualified as described in Ahmad et al.^37^ For domain presence, NCBI CDD (https://www.ncbi.nlm.nih.gov/Structure/bwrpsb/bwrpsb.cgi) and SMART database (http://smart.embl-heidelberg.de) were used to validate P450 domain.^39^ Genome organization and chromosome location information of CYPs were retrieved from the Rice Genome Database of China (http://btn.genomics.org.cn/rice) and Rice Genome Project (RGP, http://rgp.dna.affrc.go.jp). Protein characteristics feature such as molecular weight (MW) and pI (isoelectric point) were assessed in Sequence Manipulation Suite (SMS V2, http://www.bioinformatics.org/sms2/index.html); *in-silico* subcellular location prediction was analyzed using WOLFPSORT (https://wolfpsort.hgc.jp/), and MapDraw^40^ was used to display each gene on the chromosome. The structure of CYP genes was visualized in the gene structure display server (GSDS, http://gsds.gao-lab.org/index.php). For *cis*-regulatory elements prediction, 1000 bp promoter sequences of each gene from the transcription start site were retrieved from RGP and analyzed in NEW PLACE (https://www.dna.affrc.go.jp/PLACE/?action=newplace).

### Phylogeny of CYPs in Rice and Arabidopsis

The phylogenetic relationships among the rice and Arabidopsis CYPs were analyzed in MEGA7 to generate an unrooted neighbor-joining (NJ) phylogenetic tree described by Wei et al.,^38^ Arabidopsis CYP protein sequences were retrieved from TAIR (http://www.arabidopsis.org/index.jsp) and aligned in ClustalOmega (https://www.ebi.ac.uk/Tools/msa/clustalo/) and finally submitted in MEGA7.^41^ The bootstrap was set at 1000 replicates.

### Plant Growth Conditions and Material Collection

The surface-sterilized seeds of Japonica rice (*O. sativa* cv. Zhonghua11) were grown under conditions described in Ahmad et al.^37^ The fresh samples of different plant parts, such as stem, root, leaf, node, and panicle, were harvested. For expression analysis of CYPs under various stresses, such as drought, salinity, and phytohormones, including indole-3-acetic acid (IAA), abscisic acid (ABA), and gibberellin (GA), two-week-old rice seedlings were used following as described in Ahmad et al.^37^ Samples were collected at 0 h, 1 h, 3 h, 6 h, 12 h, and 24 h after post-treatments.

### cDNA Synthesis and Quantitative Real-time PCR (Qrt-PCR) Assay

Trizol reagent (Invitrogen, USA) was used for total RNA extraction from harvested samples following the manufacturer’s guidelines. DNase1 (Takara, Japan) was used to remove genomic DNA contamination from samples. RNA was further purified by precipitating the digested DNA. About 2 μg of total RNA was used for cDNA synthesis in the RT-PCR system (Promega, USA) following the manufacturer’s instructions. To perform qRT-PCR of selected genes, SYBR Green Master Mix (Toyobo, Japan) was used in ABI-7500 Fast Real-Time PCR (Applied Biosystems, USA). QuantPrime tool^42^ was used to design gene-specific primers (Table S1). However, 18s-rRNA was used to normalized gene expression. The expression was estimated by adopting ∆Ct method as described in Jain et al.^43^

### Statistical Analysis

GraphPad prism was used for statistical analysis. Three biological replicates for each sample were used for RT-qPCR analysis and five technical replicates were analyzed for each biological replicate. To determine significant differences two-tailed Student’s *t*-tests was used at *P* values (*P < .05; ** *P* < .01; *** *P* < .001; **** *P* < .0001).

## Results

### Identification and Characterization of Methylation Regulated Candidate CYP Genes

**Figure 1.**
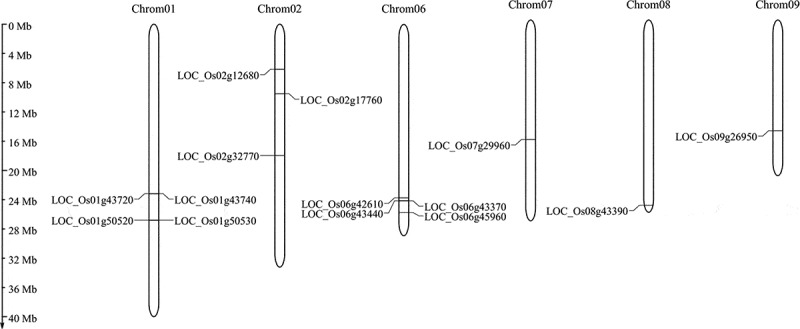
Chromosomal distribution of rice OsCYPs in the rice genome. The scale on the right side is corresponding to the length of each chromosome.

We adopted various bioinformatics approaches to search OsCYP of the grapevine genome, including BLAST and the hidden Markov model (HMM) profile method. A total of 384 CYP genes, including those 355 CYP genes previously identified by Wei et al.,^38^ were detected and subsequently verified for the P450 domain (PF00067). Among them, 14 potential CYPs showed varying methylation levels were identified through methylated DNA immunoprecipitation (MeDIP)-chip hybridization in rice under salt and osmotic stress. The protein length of methylation regulated OsCYPs ranging from 106 aa (LOC_Os09g26950) to 551 aa (LOC_Os08g43390). Table 1 shows the additional information about OsCYPs including their peptide length, pI, MW, in-silico subcellular location, and genome distribution. According to gene mapping, 14 OsCYPs were anchored on six chromosomes in the rice genome (Fig. 1). Chromosomes 1 and 6 anchored four genes each, three on chromosome 2, and a single gene was found on chromosomes 7, 8, and 9. Moreover, the number of exons varies from one to nine among 14 methylations regulated OsCYPs. For example, the single exon is found in LOC_Os09g26950 and LOC_Os02g12680. However, LOC_Os07g29960 and LOC_Os01g43740 displayed nine and five exons each whereas, the rest of the genes contained two exons except for LOC_Os02g17760, which contained three (Figure S1).

**Table 1.** Characteristics of OsCYPs proteins in rice under

Gene ID	Gene Putative Function	aa	MW	pI	In silico subcellular location	Chromosome		
						Number	Start	End
LOC_Os01g43720	cytochrome P450 72A1, putative, expressed	381	43.40	9.10	Chlo	1	25058195	25055772
LOC_Os01g43740	cytochrome P450 72A1, putative, expressed	535	60.20	7.82	Cyto	1	25060764	25063609
LOC_Os01g50520	cytochrome P450 CYP711A12, putative, expressed	128	14.20	10.17	Chlo	1	29020844	29021337
LOC_Os01g50530	cytochrome P450, putative, expressed	412	46.79	8.71	Chyto	1	29024351	29025826
LOC_Os02g12680	cytochrome P450, putative, expressed	500	54.39	7.01	Chlo	2	6628383	6630103
LOC_Os02g17760	cytochrome P45071D8, putative, expressed	540	59.13	8.12	Chlo	2	10261986	10268672
LOC_Os02g32770	cytochrome P450, putative, expressed	508	56.34	8.48	Chlo	2	19463361	19459069
LOC_Os06g42610	cytochrome P450, putative, expressed	254	27.48	9.73	Chlo	6	25620940	25622315
LOC_Os06g43440	cytochrome P450, putative, expressed	220	24.50	4.95	Cysk	6	26109554	26108626
LOC_Os06g43370	cytochrome P45071D10, putative, expressed	513	55.46	8.40	Chlo	6	26072378	26074146
LOC_Os06g45960	cytochrome P450, putative, expressed	547	50.56	9.04	Chlo	6	27848256	27844654
LOC_Os07g29960	cytochrome P450 87A3, putative, expressed	405	46.68	6.64	Chlo	7	17643252	17640723
LOC_Os08g43390	cytochrome P45078A3, putative, expressed	551	59.8	9.38	Chlo	8	27422836	27420501
LOC_Os09g26950	cytochrome P450 CYP92A1, putative	106	12.09	11.95	Chlo	9	16377280	16376963

### Phylogenetic and Motif Analysis of Rice CYPs

**Figure 2.**
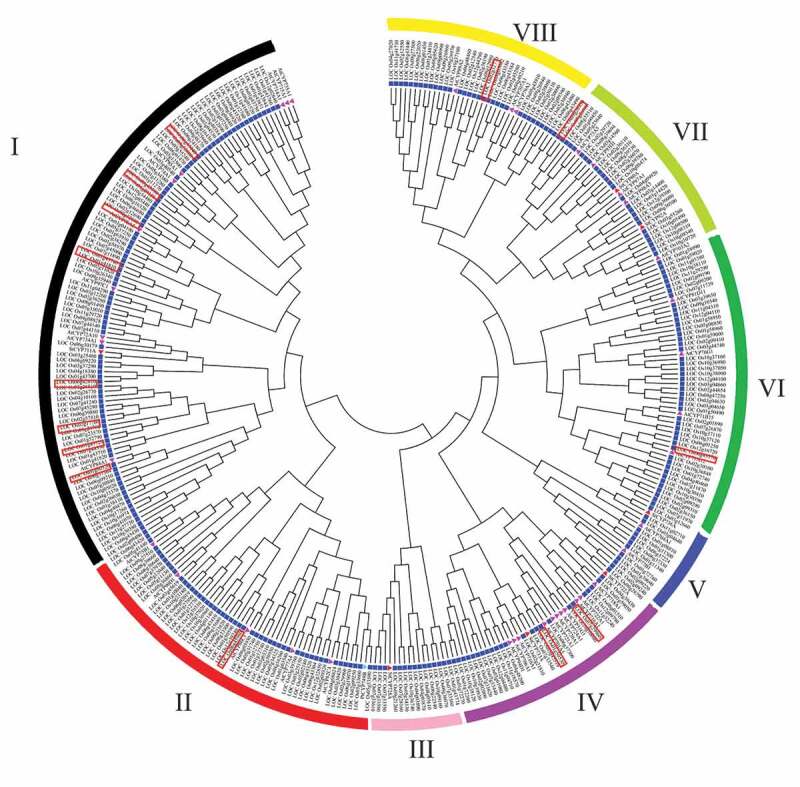
Phylogeny of CYPs in different plant species. An unrooted phylogenetic tree was generated with 1,000 bootstrap replicates and a *p*-distance method in the MEGA7 program using protein-coding sequences of CYPs from Arabidopsis, *Solanum tuberosum*, and *Populus trichocarpa.*

To determine the phylogenetic relationship of OsCYP genes with CYPs from Arabidopsis, *Solanum tuberosum*, and *P. trichocarpa*, an unrooted NJ phylogenetic tree was generating. All CYPs were clustered in 9 clades (Fig. 2) and rice methylation regulated CYPs grouped in 6 different clans in the phylogenetic tree along with other OsCYPs (Fig. 2). Eight OsCYPs were clustered in Group-I, followed by two OsCYPS in cluster IV. Moreover, group II, VI, VII, and VIII contained a single rice protein. MEME motifs analysis revealed five unique motifs in 12 OsCYPs but absent in LOC_Os01g50520 and LOC_Os09g26950, while LOC_Os01g43720 contained three motifs only. LOC_Os02g12680 and LOC_Os01g43740 contained motif 2/4 and motif 1/3/4, respectively. LOC_Os07g29960, LOC_Os06g43440, LOC_Os06g42610, and LOC_Os01g50530 showed motif 1/2/3/4 in their protein sequences. The rest of the OsCYPs contained all motifs in their peptides (Figure S2).

### DNA Methylation Status and Correlation Analysis of Candidate CYPs in Rice

By analyzing microarray data in three different conditions, 14 unique P450 genes exhibiting co-ordinately change in DNA methylation and expression were identified. To analyze whether the genes significantly affected epigenetically (>2-fold changes in methylation and expression) or not, a variable trend of methylation under two comparative conditions (control vs. stressed) was assayed. Here, an average normalized depth of reads as the measurement of methylation level for each gene was used. To quantify methylation level in control, salt, and osmotically stressed samples, we used MEDIPS software package.^44^ To quantify DNA methylation in genes, all the CYPs were divided into different basic genic regions like a promoter, gene body, 5′ UTR, 3′ UTR, and downstream. Genome-scale peak scanning (unique peaks) for methylation was based on behalf of a defined analysis model (MACS), and the peak standard was fixed on behalf of *P*-value (*p* < 10*e*-5). Peaks of two samples (control vs. stressed) were merged as candidate different methylation regions (DMRs). Genomic regions with at least three consecutive windows statistically significant differentially methylated samples were considered differentially methylated regions (DMRs).

Overall, we observed a wide range of methylation differences in CYPs under two comparative conditions, and the other was predicted in the promoters of the basic region of methylation, and out of 14 genes, nine were methylated in the promoter region. After promoter, downstream and gene body (CDS) were highly methylated regions among CYPs. Different quantities and varying levels of methylation in different regions represent the patterns of methylation, and the changed level of methylation might be the cause for their up/downregulation. Among CYPs genes, we also observed the enrichment of methylation in different genes under two different stresses (control vs. salt or PEG6000 stress). LOC_Os02g12680 showed the methylation quantity in upstream 2 K and downstream 2 K region and showed down-regulation under both stresses. Similarly, LOC_Os06g42610 showed the enrichment of methylation in downstream 2 K, CDS, and intron regions and downregulated under salt and osmotic stresses. Another gene, LOC_Os09g26950, was methylated in upstream 2 K and CDs regions.

**Figure 3.**
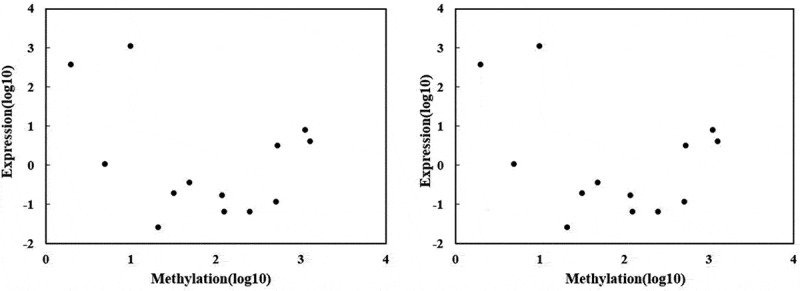
Correlation analysis between DNA methylation and genes (CYPs) expression (a) correlation under salt stress (b) correlation between DNA methylation and gene expression under osmotic stress. The X-axis indicates the level of methylation, while Y-axis is the gene’s expression. Each point in the graph represents a specific gene of P450.

Two different natures of stresses cause different changes epigenetically in the rice genome through the up/downregulation of genes. For example, the gene LOC_Os08g43390 showed its maximum DNA methylation 530 under salt stress compared to osmotic stress, where its methylation quantity was 444. Similarly, another gene, LOC_Os06g42610, showed 1125 quantities of methylation under salt stress and 1052 under osmotic stress, while its level of methylation was only 457 under control for downregulation. Another CYP gene (LOC_Os02g12680) was less methylated (98) under osmotic stress compared to salt stress (119). The detailed information of CYP genes about their methylation status and correlation under two comparative conditions is given in Table 2 and Fig. 3a–b.

**Table 2.** Methylation (5mC) status of identified P450s under salt and osmotic stresses in rice

	Gene ID	Stress regulation	Specific site of methylation	Specific region of methylation	DNA (Cytosine-5) methylation data			Up/down regulated	Up/down regulated	
					CK (control)	Salt stress	PEG6000 stress	under salt stress	Under Osmotic stress	
	LOC_Os01g43720	Salt, Osmotic	25054728–25054728	**Upstream2K**	30	21	28	Down regulated	Up regulated	
	LOC_Os01g43740	Salt, Osmotic	25059720–25062565	**Upstream2K**	68	49	85	Down regulated	Up regulated	
	LOC_Os01g50520	Salt, Osmotic	29019799–29020292	**Downstream2K**	4	0	9	Down regulated	Up regulated	
	LOC_Os01g50530	Salt, Osmotic	29023306–29024781	**Upstream2K**	17	11	19	Down regulated	Up regulated	
	LOC_Os02g12680	Salt, Osmotic	6628383–6630103	**Upstream2K, Downstream2K**	118	119	98	Down regulated	Down regulated	
	LOC_Os02g17760	Salt, Osmotic	10261984–10268670	**Intron**	1106	1285	1286	Down regulated	Up regulated	
	LOC_Os02g32770	Salt, Osmotic	19453201–19457493	**Upstream2K**	182	126	172	Down regulated	Up regulated	
	LOC_Os06g42610	Salt, Osmotic	25619943–25621318	**Downstream2K, CDS, Intron**	457	1125	1052	Down regulated	Down regulated	
	LOC_Os06g43440	Salt, Osmotic	26107629–26108557	**Upstream2K**	198	252	237	Down regulated	Down regulated	
	LOC_Os06g43370	Salt, Osmotic	26071381–26073149	**Upstream2K**	44	32	44	Down regulated	Up regulated	
	LOC_Os06g45960	Salt, Osmotic	27843656–27847258	**Upstream2K**	778	514	520	Up regulated	Up regulated	
	LOC_Os07g29960	Salt, Osmotic	17639730–17642259	**Downstream2K**	27	19	28	Up regulated	Up regulated	
	LOC_Os08g43390	Salt, Osmotic	27417787–27420122	**CDS, 3ʹUTR**	180	530	444	Down regulated	Up regulated	
	LOC_Os09g26950	Salt, Osmotic	16375962–16376279	**Upstream2K, CDS**	98	275	256	Down regulated	Up regulated

### *Expression Analysis of* OsCYPs *in Different Parts/organs of the Rice Plant*

**Figure 4.**
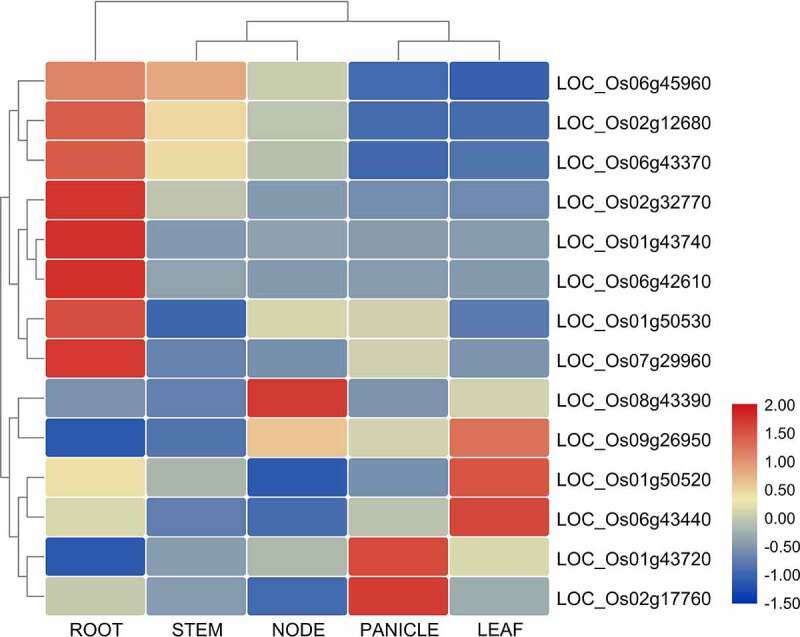
Heatmap of tissue-specific expression of CYPs in different parts of the rice plant, including root, stem, leaf, panicles, and node. All the values are log^2^ transformed. The red and blue color is corresponding to high and low expressions.

The specific expression pattern of methylation-regulated OsCYPs was analyzed by qRT-PCR in different plant parts including, stem, root, node, panicle, and leaf. It was observed that OsCYPs are highly specific in their expression pattern (Fig. 4) which support the idea of tissue-specific epigenetic changes and the regulation of different CYPs under abiotic stresses. LOC_Os06g45960, LOC_Os02g12680, LOC_Os06g43370, LOC_Os02g32770, LOC_Os01g43740, LOC_Os06g42610, LOC_Os01g50530, and LOC_Os07g299660 were highly expressed in root than other tissues. LOC_Os08g43390 expression peaked in nodes, while LOC_Os09g26950 have a weak expression in node but showed strong leaf expression. Similarly, LOC_Os01g50520 and LOC_Os06g43440 peaked in leaf, but the former has a weak expression in root. LOC_Os02g17760 and LOC_Os01g43720 were highly expressed in a panicle (Fig. 4).

### OsCYPs Promoter Characteristic Features

Furthermore, for the distribution of *cis*-acting elements, OsCYP genes promoter sequences were analyzed. The promoter regions of OsCYPs contain a variety of *cis*-acting elements such as those for abiotic stress include, ABRE (for abscisic acid responsiveness), TGACG-motif (for MeJA responsiveness), ERE (Ethylene responsive), LTR (for low-temperature responsiveness), TATC/GARE (gibberellin-responsive), TCA-Element (for salicylic acid-responsiveness), MBS (drought-inducibility), TGA-Element (Auxin responsive), and AuxRR-Core (auxin responsiveness) (Table S2).

### Phytohormone Induced Expression Patterns of OsCYPs

**Figure 5.**
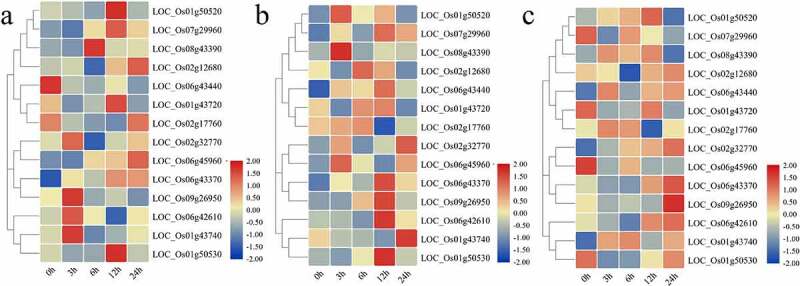
Heatmap of expression patterns of 14 OsCYPs under (a) abscisic acid (ABA), (b) indole-3-acetic acid (IAA), and (c) gibberellins (GA_3_) at 0 h, 3 h, 6 h, 12 h, and 24 h after treatment. The expression was determined relative to expression at 0 h. The red and blue color is corresponding to high and low expressions. All the values are log^2^ transformed.

Numerous *cis*-regulatory sequences related to hormone stresses were identified in promoter sequences of OsCYPs (Table S2). Thus, we examined the phytohormones-induced expression profile of these genes in the rice plant, including root and shoot (Fig. 5). The hormone-induced expression profile analysis revealed that few methylation-regulated rice CYPs genes were highly sensitive, while some of them were insensitive. LOC_Os06g43370, LOC_Os06g45960, LOC_Os07g29960, and LOC_Os08g43390 showed temporal expression at all time intervals. It was found that the promoters of these genes had *cis*-regulatory elements related to ABA response (ABRE) (Table S2). LOC_Os01g43740, LOC_Os02g32770, LOC_Os09g26950, and LOC_Os06g42610 showed peaked expression at three hours. However, LOC_Os08g43390 at six h; LOC_Os01g43720 and LOC_Os01g50520 at 12 h; LOC_Os02g17760 at 24 h were upregulated (Fig. 5a). For IAA treatment, LOC_Os01g50520, LOC_Os08g43390, and LOC_Os06g45960 were upregulated at three hours but downregulated during later time intervals. LOC_Os01g50530, LOC_Os06g43370, LOC_Os07g29960, LOC_Os09g26950, and LOC_Os06g42610 peaked at 12 h after IAA exposure. Similarly, LOC_Os01g43740 and LOC_Os02g32770 were upregulated at 24 h after treatment (Fig. 5b). Similarly, we observe that only of these genes’ promoter contained *cis*-regulatory elements related to IAA among IAA-activated genes. These genes include LOC_Os02g32770 LOC_Os06g42610, LOC_Os06g45960, LOC_Os07g29960, and LOC_Os08g43390 (Table S2). For GA treatment, LOC_Os01g43720, LOC_Os07g29960, and LOC_Os06g45960 were downregulated after treatment. LOC_Os01g43740, LOC_Os08g43390, LOC_Os01g50520, and LOC_Os02g32770 were upregulated all time intervals except for LOC_Os08g43390 and LOC_Os01g50520, which was downregulated at 24 h after treatment. It is worth mentioning that only the promoter of LOC_Os01g50520 contained gibberellin-responsive elements (Table S2), suggesting that other regulatory genes may interact with these CYPs to activate their response to GA. However, LOC_Os06g42610 and LOC_Os09g26950 peaked at 24 h intervals (Fig. 5c). It is interesting to note that few genes were upregulated under all stresses while others induced under one of three stresses such as LOC_Os02g32770, LOC_Os06g42610, LOC_Os06g4337, and LOC_Os08g43390 was induced under all stresses whereas LOC_Os01g43720 was induced under IAA, but LOC_Os01g43740 was suppressed. The phytohormones-induced expression of OsCYPs suggested that these stresses might regulate their expression pattern.

### Expression Analysis of Rice Methylation Regulated CYPs Genes under Salt and PEG Stresses

**Figure 6.**
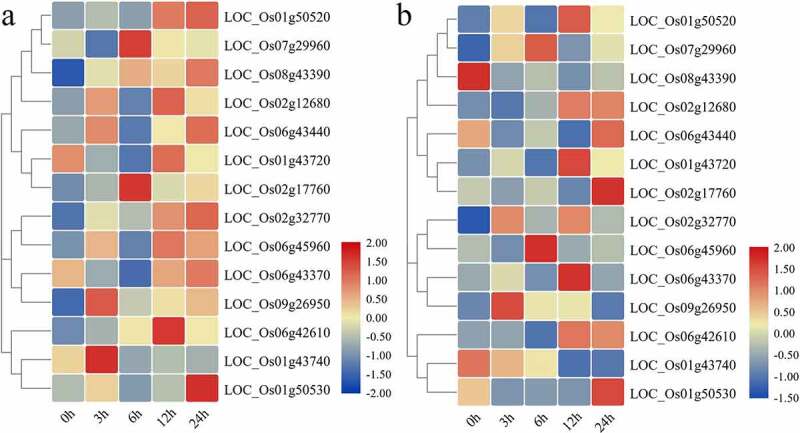
Heatmap of expression patterns of 14 OsCYPs under (a) salinity (b) Drought stresses at 0 h, 3 h, 6 h, 12 h, and 24 h after treatment. All the values are log^2^ transformed. The expression was determined relative to expression at 0 h. The red and blue color is corresponding to high and low expressions.

To study the transcriptional regulation of OsCYPs under salinity and drought, we also analyzed the *cis*-acting elements in the promoters of CYPs. It was predicted that CYP genes contain *cis*-acting motifs such as temperature (LTR), drought (MBS), light (G-Box), and the pathogens may respond to abiotic stresses (Table S2). To understand the salinity and drought-induced expression profile of OsCYPs, the expression of 14 methylation-regulated genes was profiled. It was observed that LOC_Os01g43740 and LOC_Os09g26950 induced at 3 h; LOC_Os02g17760 and LOC_Os07g29960 at 6 h; LOC_Os06g42610 at 12 h; LOC_Os01g50530 at 24 h under salt stress. LOC_Os01g50520, LOC_Os02g32770, and LOC_Os08g43390 were upregulated at all time intervals and showed peaked expression at 24 h after stress (Fig. 6a). For drought, LOC_Os01g43740 and LOC_Os08g43390 were downregulated, but LOC_Os01g50530 and LOC_Os02g17760 peaked at 24 h. LOC_Os02g32770 and LOC_Os09g26950 upregulated at 3 h; however, LOC_Os07g29960 was peaked at 6 h; LOC_Os01g50520 and LOC_Os06g43370 at 12 h; LOC_Os02g17760 was significantly peaked at 24 h (Fig. 6b). Overall, some genes were upregulated or downregulated under both stresses, while few showed opposite trends. LOC_Os01g43720 and LOC_Os06g45960 were downregulated under salinity, and LOC_Os02g17760, LOC_Os06g42610, LOC_Os06g43370, and LOC_Os06g45960 were suppressed under drought. LOC_Os01g50530, LOC_Os06g43440, and LOC_Os06g43370 were downregulated under both stresses.

#### Discussion

DNA methylation (5mC) plays a dynamic role in regulating plant development, helping plant adaptation to environmental changes.^45–47^ The mechanism by which DNA-methylation participates in these complex processes and mechanism is still unclear. The salinity and drought demethylate their responsive transcription factors, such as WRKY, NAC, TCP, and ARF, have been reported as key transcriptional regulators under stress conditions on behalf of DNA-methylation.^48–50^ *Nicotiana tabacum* DNA-methyltransferase 1 (*met1*) mutant removes methyl-group from various genomics regions, activating stress-responsive genes.^51^ However, the general mechanism of DNA-methylation associated with these stress-responsive genes remains unknown. Genome-scale DNA-methylation analysis was performed in various organisms from insects, chordates, animals to plants.^52,53^ This revealed that it might be considered as the highlighted feature of all eukaryotes.^54^

Rice is an important cereal crop worldwide, and a facile genomic model provides an opportunity to study the molecular biology of cereal plants. Rice DNA-methylation is of great interest to geneticists, biotechnologists, and breeders. We observed a significant enrichment of upregulated and downregulated genes associated with DNA-methylation or demethylation in rice. It was also analyzed that a large fraction of DEGs (differentially expressed genes) did not exhibit a significant difference in their methylation level (Table 2). Different researchers reported similar findings describing the role of DNA-methylation in transcriptional regulation of specific loci in different crops.^51,55,56^ Genome analysis of these genes indicated that DNA-methylation plays a pivotal role in regulating hormone-specific CYP genes. A strong correlation of gene promotor and body methylation/demethylation for genes up/downregulation compared to any other flanking sequence is an essential feature of stress-responsive genes like CYPs to support the plant the adaptation of environmental stress like salt and drought. These findings are consistent with previous reports, including rice.^6,48,57,58^

In the current study, the DNA-methylation in CYPs is estimated for the first time through genome analysis. Changes in the DNA-methylation level of CYPs and other related regulatory genes are the key to future crop breeding programs. Rice, being a salt-sensitive cereal crop, displayed high genotypic variability.^59,60^ These genotypes can tolerate extreme salinity, while few are highly sensitive. These are indicating that the unique rice genetics and regulatory mechanism are essential for further improvement. This study identified a unique array of genes, including stress tolerance, chromatin modification, retrotransposons, and hormone responsive. It suggests that the evolution of natural variability in rice germplasm for stress tolerance might be independent of the patterns or extent of DNA-methylation. Wang et al.^47^ unveiled that DNA-methylation variation exhibited tissue specificity and caused significant differential gene expression. In tissues-specific expression analysis, we also find a diverse and variable expression of P450 genes in rice tissues that all genes are constitutively expressed (Fig. 4). Finally, to demonstrate transcription regulation, the cis-acting sequences analysis revealed that OsCYP genes respond to various abiotic stresses such as abscisic acid, MeJA, and ethylene low-temperature, gibberellin, salicylic acid, drought, auxin (Table S2).

## Conclusion

Conclusively, a dynamic correlation was observed between DNA methylation and CYP genes’ expression under abiotic stresses by high throughput analysis. Varying level of DNA-methylation was observed in different regions of concerned genes under two comparative conditions. This may show their regulatory role in rice plants against these stresses and predicts the epigenetic role of DNA-methylation in gene suppression or activation. This study provides the foundation to explore the enzymatic activity of CYPs and find out the impact of DNA-methylation in gene stress adaptation mechanisms for rice and other related crops to improve growth and yield as well.

## Supplementary Material

Supplemental Material
