# Supplementary material for: Identification, methylation profiling, and expression analysis of stress-responsive cytochrome P450 genes in rice under abiotic and phytohormones stresses
Source: GM Crops Food. 2021 Apr 20;12(1):551–63. doi: 10.1080/21645698.2021.1908813 (PMC8820252; doi:10.1080/21645698.2021.1908813)
Supplement: Supplemental Material [file KGMC_A_1908813_SM0468.docx]

**Identification, methylation profiling and expression analysis of stress-responsive cytochrome P450 (P450) genes in rice under abiotic and phytohormones stresses**

Muhammad Waseem^1†^, Feiyan Huang^2*†^, Mehtab Muhammad Aslam^3^, Farhat Abbas^4^, Fiaz Ahmad^5^, Umair Ashraf^6^, Waseem Hassan^7^, Lei Yu^2^, Qiyu Wang^2^, Yanguo Ke^8*^

^1^College of Horticulture, South China Agricultural University, P.R. China

^2^College of Agriculture and life sciences, Yunnan Urban Agricultural Engineering & Technological Research Centre, Kunming University, Kunming 650214, China

^3^College of Life Sciences, Joint International Research Laboratory of Water and 5 Nutrient in Cops, Fujian Agriculture and Forestry University, Fuzhou, Fujian 350002, China

^4^College of Forestry and landscape architecture, South China Agricultural University, P.R. China

^5^ State key Laboratory of Crop Genetics and Germplasm Enhancement, Nanjing Agricultural

University, Nanjing 210095, PR China

^6^Department of Botany, Division of Science and Technology, University of Education Lahore, 54770, Punjab, Pakistan

^7^Institiute of Environment and Sustainable Development in Agricultural, Chinese Academy of Agricultural Sciences, 100081, Beijing, China

^8^College of Economics and Management, Kunming university, Kunming 650214, China

**Supplementary data**


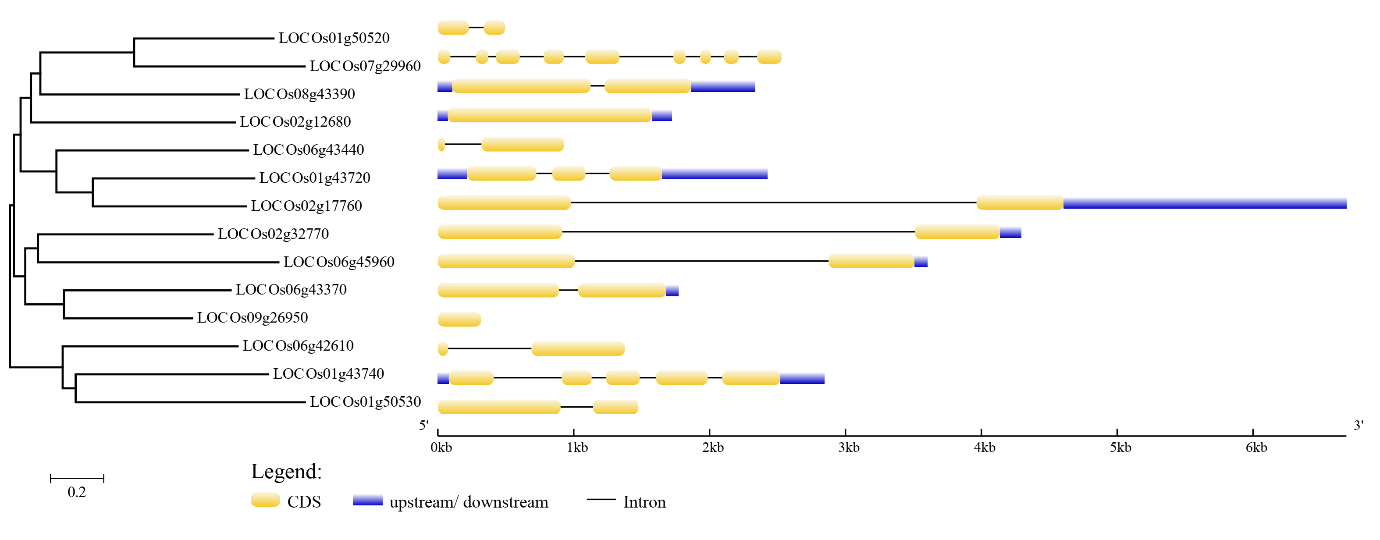


Figure S1. Exon-intron distribution of methylation regulated rice CYPs predicted in GSDS server using coding and genomic sequences of corresponding genes.


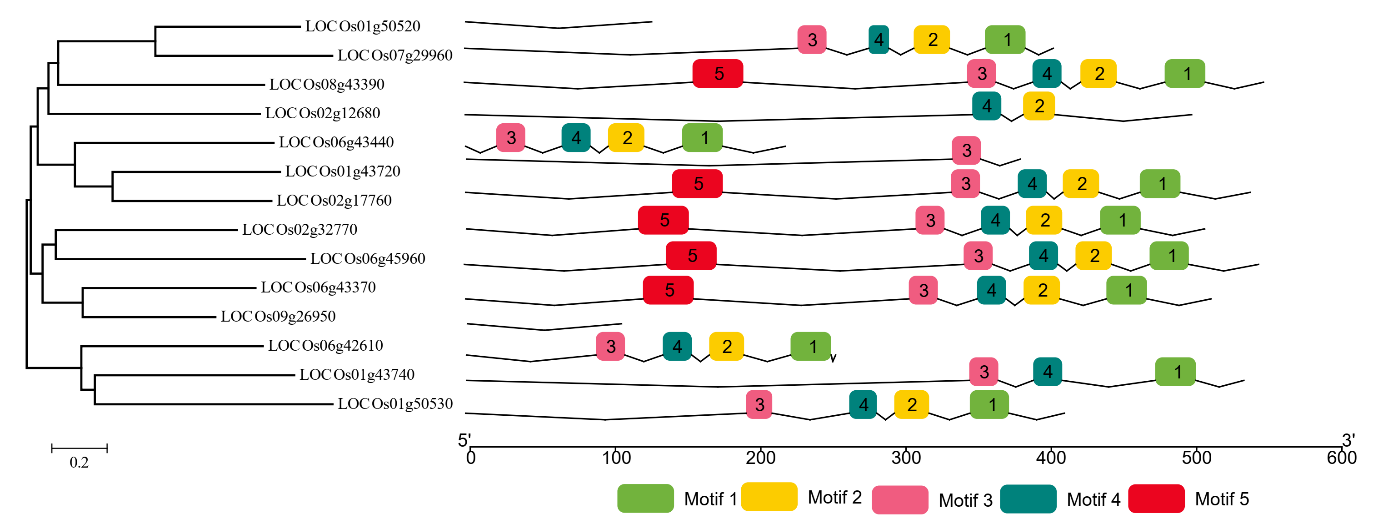


Figure S2. MEME motif analysis in methylation regulated rice CYPs.

Table S1. List of primers used in this study

| **S. NO.** | **Gene ID** | **Forward primer (5'-3')** | **Reverse Primer (5'-3')** |
| --- | --- | --- | --- |
| **1** | LOC_Os01g43720 | AGGCCTGAGCAAACTCAAAA | ACAAGCGGAGAACCTCGTAA |
| **2** | LOC_Os01g43740 | GGGAGTCTCACTGGAAGCAG | GGCGTTCTCCGGTAGGTC |
| **3** | LOC_Os01g50520 | CGAGCCATACTGGAAAGTGC | TCCTTGCACAACTCAGCATC |
| **4** | LOC_Os01g50530 | CAGGTTCTCAAGAGGCTTCG | GGAGGTGCTCGTAAGTGAGC |
| **5** | LOC_Os02g12680 | GGAGCTACTCCTGCACACCT | TGTGGCAGGAAGATCTTGAA |
| **6** | LOC_Os02g17760 | TGGCATCGACATTAGTGAGC | CCGCATTGTCCCAGTACTTT |
| **7** | LOC_Os02g32770 | GTAGCGAGACATCAGCCACA | AGCCCATAACCTTGCATGTC |
| **8** | LOC_Os06g42610 | AGTGGAAAAGGAGTGCCAGA | CATACCGTGGACGTTCTCCT |
| **9** | LOC_Os06g43440 | GGAGCAGGACGAAGAATCTG | TACCAACAGGTCGGCTTTTC |
| **10** | LOC_Os06g43370 | CTCGGCTTAGCTCTCGTGTC | GAGCCGTAGCATCATGACC |
| **11** | LOC_Os06g45960 | TATGGGCCATCGGTAGAGAC | GGAGGAAGATGCCAATCAAA |
| **12** | LOC_Os07g29960 | GGAGCCTCACCACGTACAAT | GCACCTCGATCCTTGGTTTA |
| **13** | LOC_Os08g43390 | CAAGCGGTGATCAAAGAGGT | ACCCCATTATCGGAAACTCC |
| **14** | LOC_Os09g26950 | GTGTATAGGCTCCCGCTAGG | GTTTCTCATCCGCCATTGAT |

Table S2. Distribution of cis-acting elements in promoter sequences of rice CYPs. Cis-regulatory elements were predicted PlantCARE database by submitting -1000 bp promoter sequence of 14 CYPs.

| Cis-acting element | Sequence | Description |  |
| --- | --- | --- | --- |
| ABRE | CCT/ACGTGG/C | Cis-acting element involved in the abscisic acid responsiveness | LOC_Os01g43720 LOC_Os01g43740  LOC_Os01g50520 LOC_Os01g50530  LOC_Os02g12680 LOC_Os02g17760  LOC_Os02g32770 LOC_Os06g42610  LOC_Os06g43440 LOC_Os06g43370  LOC_Os06g45960 LOC_Os07g29960  LOC_Os08g43390 |
| TGACG-motif | TGACG | Cis-acting regulatory element involved in the MeJA-responsiveness | LOC_Os01g43720 LOC_Os01g43740  LOC_Os01g50520 LOC_Os01g50530  LOC_Os02g12680 LOC_Os02g17760  LOC_Os02g32770 LOC_Os06g42610  LOC_Os06g43440 LOC_Os06g43370  LOC_Os06g45960 LOC_Os08g43390  LOC_Os09g26950 |
| ERE | ATTTCAAA | Ethylene-responsive element | LOC_Os01g50520 LOC_Os01g50530 LOC_Os02g32770 LOC_Os06g43440  LOC_Os06g43370 LOC_Os09g26950 |
| LTR | CCGAAA | Cis-acting element involved in low-temperature responsiveness | LOC_Os01g50520 LOC_Os01g50530  LOC_Os02g12680 LOC_Os02g17760 |
| TATC/GARE | GC/CTTTTG/AGT | gibberellin-responsive element | LOC_Os01g50520 LOC_Os01g50530  LOC_Os02g12680 LOC_Os06g43440  LOC_Os06g43370 LOC_Os09g26950 |
| TCA-Element | GAGAAGAATA | Cis-acting element involved in salicylic acid responsiveness | LOC_Os01g50520 LOC_Os01g50530  LOC_Os02g17760 LOC_Os06g43440  LOC_Os06g45960 LOC_Os07g29960  LOC_Os08g43390 |
| MBS | TAACTG | MYB binding Site/MYB binding site involved in drought-inducibility | LOC_Os01g43720 LOC_Os02g32770  LOC_Os06g43370 LOC_Os08g43390  LOC_Os09g26950 |
| TGA-Element | AACGAC | Auxin-responsive element | LOC_Os02g12680 LOC_Os02g17760  LOC_Os02g32770 LOC_Os06g42610  LOC_Os06g43440 LOC_Os06g45960  LOC_Os07g29960 LOC_Os08g43390 |
| AuxRR-Core | GGTCCAT | cis-acting regulatory element involved in auxin responsiveness | LOC_Os02g12680 LOC_Os02g17760 |
